# Case Report: Botulism associated with cosmetic BoNT-A injections: respiratory failure and multidrug-resistant infection as emerging clinical challenges

**DOI:** 10.3389/fphar.2025.1680693

**Published:** 2025-10-14

**Authors:** Yiming Song, Jinyu Li, Yujiao Ren, Wei Zhang, Hongbo Liu, Zhenyi Li, Jiarui Xu, Xingguo Zhang, Dongxing Liu, Yi Zhou, Baobao Feng

**Affiliations:** ^1^ Department of Emergency, Shandong Provincial Hospital Affiliated to Shandong First Medical University, Jinan, China; ^2^ Medical Department, Linyi People’s Hospital, Linyi, China; ^3^ Department of Poisoning and Occupational Diseases, Shandong Provincial Hospital Affiliated to Shandong First Medical University, Jinan, China; ^4^ Department of Critical Care Medicine, Shandong Public Health Clinical Center, Jinan, China; ^5^ Department of Emergency, Linyi Central Hospital, Linyi, China

**Keywords:** respiratory failure, mechanical ventilation, multi-drug resistant bacteria, botulism, botulinum neurotoxin type A

## Abstract

Botulism is a life-threatening neurotoxin-mediated disease characterized by flaccid descending paralysis which begins with cranial nerve palsies and might progress to extremity weakness and respiratory failure. Respiratory failure following cosmetic injections were scarcely reported due to the low dose of botulinum neurotoxin type A (BoNT-A) used. We present two cases of respiratory failure and multi-drug resistant (MDR) bacteria infection resulted from botulism following cosmetic injections. A 24-year-old female developed symptoms of dysphagia, blurred vision and dysarthria 6 days after cosmetic injections of BoNT-A, and progressed to respiratory failure which needed invasive mechanical ventilation 5 days later. Another 46-year-old female developed dizziness, headache and nausea 30 min after cosmetic injections of BoNT-A. Symptoms including ptosis, blurred vision, dysphagia, and slurred speech appeared 1 week later. The patient received mechanical ventilation due to the deteriorated respiratory failure. Both patients developed multi-drug resistant (MDR) bacteria infection during hospitalization. Though the pneumonia improved with effective antibiotics treatment, they underwent tracheostomy or second endotracheal intubation several days after successful weaning from the ventilator. Fortunately, they recovered without significant sequelae left. The widespread application of BoNT-A increases the risk of severe adverse events including respiratory failure and MDR bacteria infections. These two cases highlight the importance of pulmonary management for those with severe cosmetic botulism and health authorities’ supervision on the cosmetic injections with BoNT-A in the future.

## 1 Introduction

Botulism is a life-threatening neurotoxin-mediated disease characterized by flaccid descending paralysis which begins with cranial nerve palsies and might progress to extremity weakness and respiratory failure (Rao et al., 2021). Botulinum neurotoxin, produced by the anaerobic, gram-positive bacterium *Clostridium botulinum*, inhibits acetylcholine release at the neuromuscular junction and thus causes botulism. There are several exposure routes to the neurotoxin, including ingestion of toxin (foodborne botulism), bacterial colonization of a wound (wound botulism) or the gastrointestinal tract (infant botulism), and high-concentration cosmetic or therapeutic injections of toxin (iatrogenic botulism). Neurological symptoms are similar regardless of exposure route (Rao et al., 2021).

The U.S. Food and Drug Administration approved the use of botulinum neurotoxin type A (BoNT-A) in the cosmetic field in 2002. Due to the widespread application of BoNT-A for cosmetic or therapeutic purposes, iatrogenic botulism cases increase significantly (Chertow et al., 2006; Bai et al., 2018; Ghasemi et al., 2012; Eser et al., 2024; Landau et al., 2024; Yiannakopoulou, 2015). The most commonly reported symptoms among patients with botulism were dysphagia, blurred vision, diplopia, slurred speech, dysarthria, hoarseness, gastrointestinal symptoms, dry mouth, and dyspnea. The most common signs were ptosis, ophthalmoplegia, and descending paralysis (Rao et al., 2021). Respiratory failure is the most severe manifestation of botulism, which progresses rapidly and usually need invasive mechanical ventilation. However, respiratory failure following cosmetic injections were scarcely reported due to the low dose of BoNT-A used (Chertow et al., 2006; Eser et al., 2024). Here we present two cases of respiratory failure and multi-drug resistant (MDR) bacteria infection resulted from cosmetic injections of BoNT-A.

## 2 Case description

### 2.1 Patient A

A 24-year-old female was transferred to our hospital from a municipal hospital with invasive mechanical ventilation on 18 May 2021. She received cosmetic injections of BoNT-A without detailed product information at a personal salon on May 6th, and developed dizziness on the second day, however, she didn’t seek for medical help. The patient developed symptoms including dysphagia, blurred vision and dysarthria on May 12th, and presented to the local municipal hospital. The above symptoms progressed and dyspnea appeared, then she received invasive mechanical ventilation due to decreased oxygen saturation on May 17th. Due to her severe condition, the patient was transferred to our hospital.

**FIGURE 1:**
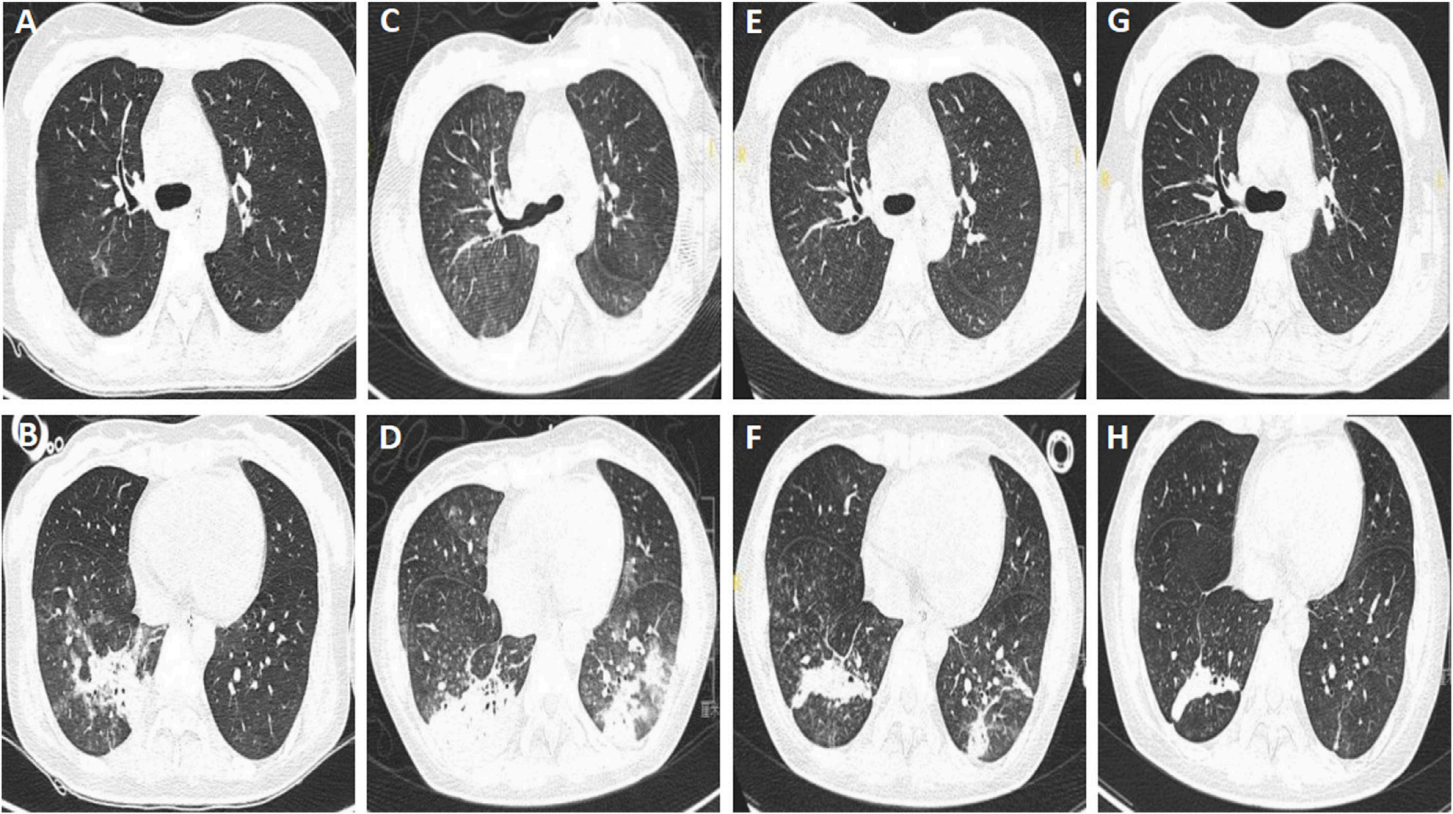
Chest computed tomography of patient A. **(A,B)** Multiple patchy ground-glass opacities and consolidations with ill-defined borders in both lungs, taken on 19 May 2021. **(C,D)** Increased infiltrating areas, taken on 2 June 2021. **(E–H)** Infiltrating areas absorbed gradually, taken on June 11th, and 20 June 2021, respectively.

The patient’s vital signs were normal on admission, except for that the muscle strength of both lower limbs were grade 4/5. Laboratory examination revealed leukocytosis (14.04 × 10^9^/L) and hypo-potassium (3.13 mmol/L), while other parameters were with normal range. The chest computed tomography (CT) taken on May 19th showed multiple patchy ground-glass opacities and consolidations with ill-defined borders in both lungs, which was consistent with aspiration pneumonia (Figures 1A,B). The patient received treatment with neostigmine, mecobalamin and vitamin B1 to improve the symptoms. She didn’t receive botulinum antitoxin injection due to the strongly positive skin test. Piperacillin tazobactam was administered to control the pneumonia at a dose of 4.5 g, every 8 h. The patient was successfully weaned from the ventilator on 26 May 2021. However, she got a fever of 38.8 °C, and productive cough with yellow phlegm on May 30th, so noninvasive ventilation was applied. Her dyspnea persisted and the partial pressure of arterial carbon dioxide (PaCO_2_) increased to 54 mmHg, therefore she received tracheostomy and mechanical ventilation on June 1st. This decision was made according to the recommendations for adult tracheostomy (Nelson et al., 2010; Lee et al., 2024). On June 2nd, the chest CT showed that the infiltrating fields increased (Figures 1C,D), and carbapenem-resistant *Enterobacter cloacae* and *Acinetobacter baumannii* were cultured from sputum specimens, then amikacin at a dose of 400 mg every 12 h was used to replace piperacillin tazobactam as per culture susceptibility test. The patient’s condition improved and she was weaned from the ventilator and received oxygen therapy at a concentration of 35% on June 8th, while the antibiotics was stopped on the same day. Chest CT were taken on June 11th, and June 20th, respectively, and the results showed that the pulmonary infection were absorbed gradually (Figures 1E–H). The tracheostomy tube was removed on June 24th, and the patient discharged with slight dysphagia left on 1 July 2021.

The symptom of dysphagia disappeared 2 months after her discharge, however, the patient has complained of limb weakness until the last followup taken on 12 March 2025, though her muscle strength was normal.

### 2.2 Patient B

A 46-year-old female was transferred to our hospital from a county hospital with invasive mechanical ventilation on 17 July 2024. She received cosmetic injections of BoNT-A by her friend at home on July 6th, and developed dizziness, headache and nausea 30 min later. She was presented to a county hospital and received neurotrophic therapy on July 8th. Symptoms including ptosis, blurred vision, dysphagia, and slurred speech developed on July 13th, followed by dyspnea on July 15th. The patient’s condition couldn’t be improved by noninvasive ventilation, so she received endotracheal intubation and mechanical ventilation on July 16th. She underwent hysterectomy 10 years ago and had a history of diabetes and hyperlipidemia for 3 years.

**FIGURE 2:**
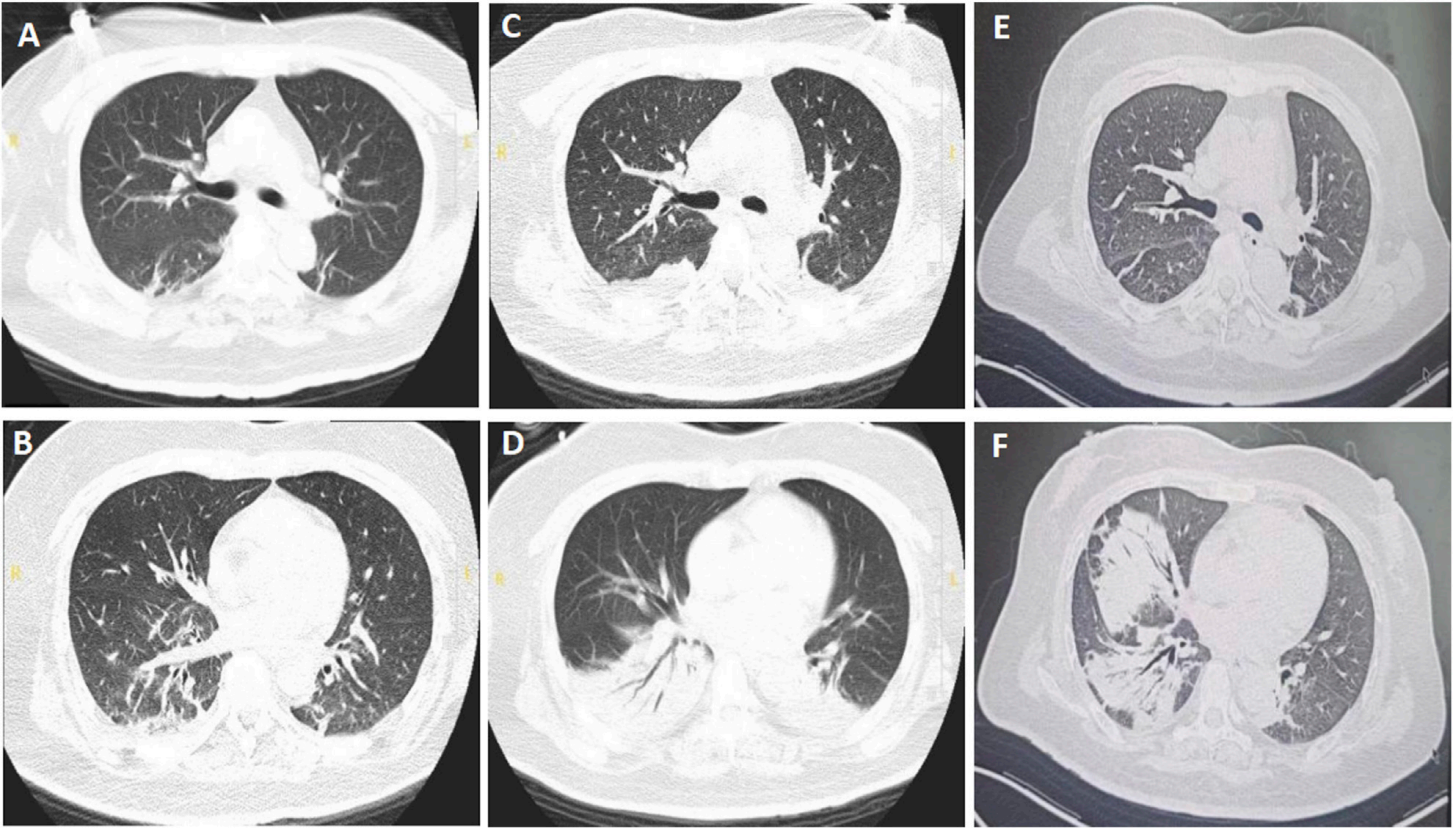
Chest computed tomography of patient B. **(A,B)** Multiple ill-defined linear and patchy opacities in both lower lobes, taken on 19 July 2024. **(C,D)** Enlarged consolidation areas of both lower lungs, taken on 29 July 2024. **(E,F)** Infiltrating fields in the left lung were partially absorbed, while those in the right lung enlarged, taken on 15 August 2024.

Upon admission, the patient had a fever of 38.4 °C and tachycardia (148 beats/min), with a normal blood pressure (106/64 mmHg). The physical examination showed moist crackles in both lungs, decreased bowel sounds, decreased muscle strength of both upper and lower limbs (grade 3/5), and reduced tendon reflexes of the extremities. Blood cytometry revealed leukocytosis (17.15 × 10^9^/L) with normal erythrocytes and platelets. Other parameters such as liver and kidney functions were normal. The chest CT taken on July 19th showed multiple ill-defined linear and patchy opacities in both lower lobes (Figures 2A,B). BoNT-A antitoxin was administered at a dose of 10,000 IU once daily. *Klebsiella pneumoniae* was cultured from sputum when the patient was hospitalized in the county hospital, so ertapenem at a dose of 1 g once daily was used to treat the pneumonia when she presented to our hospital.

The patient was successfully weaned from the ventilator at 10:20 a.m. on July 21st, and the physical examination showed that the muscle strengths of limbs were grade 4/5. Piperacillin tazobactam at a dose of 4.5 g every 8 h was used to replace ertapenem based on the normal temperature and improved leukocytosis (12.14 × 10^9^/L). However, she experienced dyspnea and difficult expectoration on the second day, with a worsened arterial gas analysis results with a pH of 7.08 and PaCO_2_ of 150 mmHg, so she was reintubated to improve the retention of CO_2_ at 22:56 on July 22nd. Due to the emergent hypercapnic respiratory failure, reintubation rather than tracheostomy was performed based on the recommendations (Nelson et al., 2010; Lee et al., 2024). On July 29th, extended-spectrum beta-lactamases (ESBLs)-producing *Klebsiella pneumoniae* was cultured from sputum, and chest CT showed that the consolidation areas of both lower lungs were significantly enlarged (Figures 2C,D), then imipenem cilastatin at a dose of 0.5 g every 6 h was used to replace piperacillin tazobactam. The patient’s condition improved thereafter and was weaned from the ventilator on July 31st. High-flow nasal cannula oxygen therapy (HFNC) was performed at a flow rate of 35 L/min and a concentration of 40%. BoNT-A antitoxin was stopped on August 1st. *Aspergillus niger* was detected from bronchoalveolar lavage fluid with a glactomannan test result of 1.47, so voriconazole at a dose of 200 mg every 12 h (the first two doses were 400 mg) was initiated on August 2nd. The patient was discharged and transferred to a respiratory specialist hospital for continued therapy based on her improved condition on August 7th.

**FIGURE 3:**
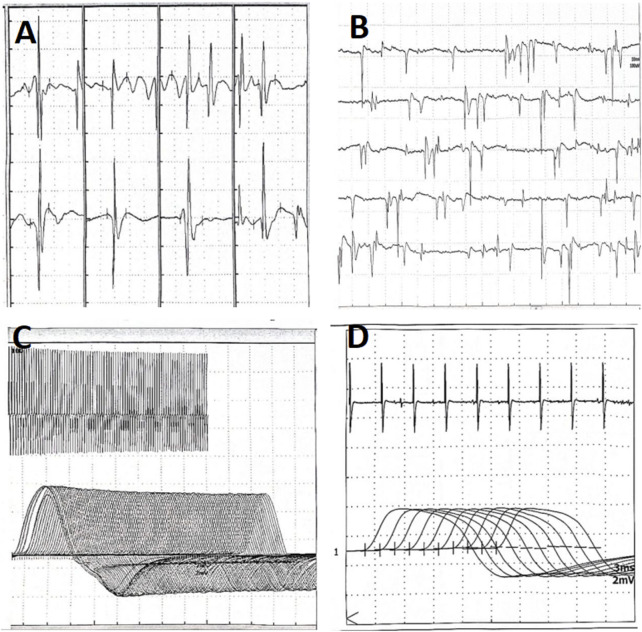
Electromyography of patient B taken on 12 September 2024. **(A)** Myogenic lesions of the quadriceps femoris. **(B)** Fibrillation and positive sharp waves of the left orbicularis oculi. **(C)** No increment on high-frequency RNS. **(D)** No decrement on low-frequency RNS. RNS, Repetitive Nerve Stimulation.

The patient received treatment of cefoperazone sulbactam and voriconazole for 2 weeks in the respiratory specialist hospital, and the chest CT taken on August 15th showed that the infiltrative fields in the left lung were partially absorbed, while those in the right lung enlarged (Figures 2E,F). She discharged from that hospital with slight productive cough and normal muscle strength of limbs. Oral voriconazole at a dose of 200 mg every 12 h was prescribed based on her improved symptoms and normal leukocyte count (4.21 × 10^9^/L). Ptosis was still present when she visited the outpatient department for review on 12 September 2024. And the electromyography showed fibrillation and positive sharp waves of the left orbicularis oculi muscle and right first interossei indicating myogenic lesions, without increment on high-frequency RNS or decrement on low-frequency RNS (Figures 3A–D). The patient finished a 3-months course of anti-fungal treatment with voriconazole without taking another chest CT. Ptosis disappeared 3 months after her discharge, and no symptoms or signs were reported when the last followup was taken on 18 March 2025.

## 3 Discussion

Foodborne botulism is the most common type which results from eating of botulinum toxin-containing foods. Infant and wound botulism develop depending on the systemic spread of the toxin after local inoculation. Cases of iatrogenic botulism have been reported since 2003, and increase significantly nowadays (Chertow et al., 2006; Bai et al., 2018; Beseler-Soto et al., 2003; Ghasemi et al., 2012; Eser et al., 2024). Iatrogenic botulism was reported to result from the use of counterfeit or unlicensed botulinum toxin products or administration of inappropriate high doses of botulinum toxin (Bai et al., 2018; Eser et al., 2024). There are eight different subtypes (A-H) of botulinum toxins, among which types A, B, and E can cause botulism in humans, while types F, G, and H cause botulism occasionally. Though the exact molecular mechanism of botulism is not clear, there has been evidence supporting the involvement of multiple steps, including heavy-chain of the toxin binding to specific receptors at presynaptic nerve terminals, internalization of the toxin into neuron and translocation to the cytosol, and cleavage of proteins necessary for neurotransmitter release by endoerepsin (Rao et al., 2021; Winner et al., 2020). Botulism is characterized by symmetric flaccid paralysis, which is the result of blockage of acetylcholine transmission across the neuromuscular junction by inhibition of acetylcholine release from the presynaptic motor neuron terminals (Humeau et al., 2000). Patients with botulism are usually alert and oriented, and rarely have sensory deficits or pain (Sobel, 2005; Shapiro et al., 1998), which are consistent with the two cases reported in this paper. Toxin type and dose, accessibility of antitoxin, and patient-level factors have been shown to have an effect on the incubation period, severity, and hospitalization of botulism (Woodruff et al., 1992; Sandrock and Murin, 2001).

BoNT-A produces the most severe symptoms, with the highest probability of respiratory failure necessitating mechanical ventilation (Chatham-Stephens et al., 2017). The lethal doses for purified crystalline BoNT-A for human beings are estimated to be 1.0 μg/kg when introduced orally and 0.011–0.013 μg/kg when inhaled (Arnon et al., 2001). For iatrogenic botulism, BoNT-A is the responsible toxin. The local effect of BoNT-A begins within 3–7 days and reaches the maximum effect on the 15th day after local injection (Moron et al., 2021). The neuron-blocking effect usually lasts for three to 6 months, and the duration of action is related to the injection site and the dose of BoNT-A (Flynn, 2010). The blockade is permanent and recovery occurs after sprouting of new nerve terminals, which usually takes weeks to months. Headache, injection site redness and edema, upper respiratory tract infection, ptosis and nausea were the most common adverse effects (AEs) (≥2%) of BoNT-A treatment (Jewell and Monheit, 2009; Naik, 2021). Dysphagia and dyspnea are serious AEs attributed to BoNT-A, which might result from the systemic spread of the toxin (Tugnoli et al., 2002; Kawashima et al., 2020; Yiannakopoulou, 2015; Maeda et al., 2023). Rare complications including morphea-like lesions, nontuberculous mycobacterial infections, pseudoaneurysm of the frontal branch of the temporal artery, necrotizing fasciitis, sarcoidal granuloma, Fournier gangrene, and cervical kyphosis were also reported (Landau et al., 2024; Yiannakopoulou, 2015).

Diagnosis of botulism is frequently delayed or even missed because botulism is much less common than other diseases with similar manifestations, such as drug or alcohol intoxication or mental status changes of other origin. The national incidence ranged from 0.00 to 8.04 cases per million people worldwide, and an estimated 88.71% of botulism cases were unreported in 2016 compared with the US standard (Learoyd, 2024). Therefore, physicians should be aware of the symptoms and signs of botulism, ranging from limited cranial nerve palsies to complete extremity paralysis and respiratory failure (Rao et al., 2021). The gold standard method to identify botulinum neurotoxin is the mouse bio-assay, which is time-consuming and need expert technicians who are experienced in recognizing botulism signs in mice. So, laboratory confirmation of botulism is usually not possible in hospital and clinical laboratories. Electrophysiological studies including repetitive nerve stimulation (RNS), electromyography (EMG), and nerve conduction study (NCS) can help distinguish botulism from muscle weakness of other origin, such as Guillain-Barré syndrome and myasthenia gravis (Rao et al., 2021).

For iatrogenic botulism, a clinical diagnosis could be made based on the medical history of therapeutic or cosmetic BoNT-A injections and typical bilateral flaccid descending paralysis. A progressive decrement on low-frequency RNS with a moderate value (<25%) supports the diagnosis of iatrogenic botulism (Theuriet et al., 2024; Ghasemi et al., 2012). Other features such as low compound muscle action potentials (CMAP), incremental responses of post-exercise CMAP, abnormal spontaneous activities, or a prolonged jitter on single-fiber EMG can be used as supportive diagnostic criteria (Theuriet et al., 2024; Ghasemi et al., 2012). These two cases didn’t take EMG during hospitalization, and the second patient’s EMG showed myogenic damage 2 months later. Ultrasound can be used to assess diaphragmatic dysfunction in patients with botulism and to predict respiratory failure that requires mechanical ventilation (Cheong et al., 2024). Negative inspiratory force can be used to monitor respiratory deterioration (Van Rynen et al., 2009).

Respiratory failure is one of the most severe symptoms and the main reason of mechanical ventilation among patients with botulism. In a systematic review consisting of 402 patients with foodborne and wound botulism, more than two fifths of the patients had respiratory involvement (shortness of breath, dyspnea, respiratory distress or failure) at admission. Patients with respiratory involvement had a shorter median incubation period (1 day; range, 0.1–12 days) than those without (1.5 days; range, 0.3–5 days) (Chatham-Stephens et al., 2017). Among the study cohort, 46% received invasive mechanical ventilation, most of whom were intubated during the first 2 hospital days. And the median duration of mechanical ventilation was 26.5 days (Chatham-Stephens et al., 2017). For the two cases in this paper, the incubation periods were within 1 day, and the symptoms progressed to respiratory failure in about 10 days. The duration of mechanical ventilation were 18 days and 14 days, respectively. Toxin types A, E, or F had greater odds of respiratory distress or failure than type B. Male, ptosis, ocular palsies, and abnormal deep tendon reflexes were significantly associated with respiratory distress or failure (Chatham-Stephens et al., 2017). Difficulty breathing, moderate to severe ptosis, and dilated and fixed pupils were associated with respiratory failure in an outbreak of food-borne botulism in Thailand (Witoonpanich et al., 2010). Literature on ventilation of iatrogenic botulism is scarce. A 6-year female with cerebral palsy developed high fever, malaise, food refusal, choking, eyelid ptosis, and absence of deep tendon reflexes after therapeutic injection of Dysport (46 U/kg). These symptoms progressed rapidly and the patients had to receive ventilatory support (Beseler-Soto et al., 2003). A 34-year old female developed respiratory failure and received invasive mechanical ventilation at 20 days after gastric BoNT-A application at a dose of 1,500 IU(Eser et al., 2024). She was successfully weaned from the ventilator 6 days later. Daniel et al. reported four cases of botulism who were injected with a highly concentrated, unlicensed preparation of botulinum toxin A with doses of 2,857 times the estimated human lethal dose by injection. All patients received mechanical ventilation for 36–171 days, and survived (Chertow et al., 2006). The two patients in this study received tracheostomy or second endotracheal intubation, 4 days and 1 day after they weaned from the ventilator. Not fully recovered muscle strength were thought to be the reasons, though they both passed the spontaneous breathing test before extubation. So it’s difficult to make recommendations on the duration of mechanical ventilation due to the scarce literature and heterogeneity among patients.

The treatment of botulism mainly consists of antitoxin therapy and intensive care including respiratory support when necessary (Karcioglu et al., 2024). To date, botulinum antitoxin was the only specific therapy for botulism, and was recommended to be administered as soon as possible (Rao et al., 2021). Early antitoxin administration could decrease the need for and duration of mechanical ventilation in patients with wound botulism (Sandrock and Murin, 2001). The most common AEs (≥1%) of botulinum antitoxin were hypersensitivity, pyrexia, tachycardia, bradycardia, anaphylaxis, and blood pressure increase (Karcioglu et al., 2024). A single randomized controlled trials demonstrated that human-derived botulinum immune globulin probably decreases the duration of hospitalization, mechanical ventilation and tube or parenteral feeding in infant intestinal botulism (Arnon et al., 2006). There are different antitoxins (mono-, tri-, or heptavalent) available worldwide, and monovalent antitoxin is the only available one in China. However, only the second patient in this paper received antitoxin, while the first didn’t due to the strongly positive skin test. In addition, the effect of antitoxin on the second patient was not ideal, since she was transferred to our hospital more than 10 days after BoNT-A injection.

Pyridostigmine can reduce the cholinergic side effects which occur after botulinum toxin applications, and clinical improvement have been observed in case reports of iatrogenic botulism (Gonul Oner et al., 2023; Ghasemi et al., 2012; Eser et al., 2024; Young and Halstead, 2014). Aminopyridines have been shown to rapidly restore ventilation and respiration, and reverse respiratory acidosis in animal models of botulism (Mcclintic et al., 2024). McNutt et al. delivered a function-blocking single-domain antibody into the neuronal cytosol by an atoxic derivative of BoNT to inhibit BoNT-A molecular toxicity. With post-symptomatic treatment, the toxic signs of botulism relieved and survival increased in animals models of three species (mice, guinea pigs, and nonhuman primates) after lethal BoNT-A (Mcnutt et al., 2021). Miyashita and colleagues developed a safe and effective post-exposure treatment for BoNT-A/B using a neuron-specific delivery platform based on a chimeric toxin approach (Miyashita et al., 2021). However, these treatments are still in pre-clinical development.

The emergence and spread of antimicrobial resistance, especially MDR have been great threat to the effective prevention and treatment of an ever-increasing range of infections caused by bacteria, parasites, viruses, and fungi. MDR was associated with longer length of stay and higher mortality rate (Sati et al., 2025). Carbapenem-resistant *acinetobacter baumannii* and ESBLs-producing *Klebsiella pneumoniae* detected from our patients were among the bacterial pathogen priority tiers recommended by the world health organization (Sati et al., 2025). Though severe respiratory failure have been reported by previous researchers, no detailed information regarding mechanical ventilation and MDR bacteria infection were demonstrated. These two cases highlight the importance of pulmonary management for those with cosmetic botulism. For patients with severe botulism, early tracheostomy might decrease the incidence of MDR, since the course of mechanical ventilation was long in this cohort. This study was limited by its small number of cases, however, it’s not realistic to carry out a large-scale research due to the low incidence of mechanical ventilation resulted from cosmetic BoNT-A injections.

Therapeutic or cosmetic BoNT-A injections are usually safe, and the most common AEs are minor or moderate. However, the huge market demand has led to the entry of inferior products with inaccurate dosage and questionable purity, and the engagement of unqualified institutions or personnel in botulinum toxin treatment, both increase the risk of severe AEs including respiratory failure and MDR bacteria infections (Bradshaw, 2017; Rashid et al., 2018; Dawson et al., 2024; Alenezi et al., 2024). A majority of patients who experienced systemic toxicity had received injections in informal settings, as the two patients in this paper. They knew little about the products information (brand, dosage, etc.) and the potential AEs. A survey revealed that only 20% of the respondents were aware of the AEs of botulinum toxin (Alenezi et al., 2024). We reported the two cases to the provincial authorities, however, no epidemiological investigation was conducted due to the low incidence. Therefore, health authorities should strengthen supervision on the cosmetic injections with BoNT-A. Measures such as prohibiting counterfeit drugs, strengthening staff training, and improving public education and information dissemination can be taken to improve the situation (Bradshaw, 2017; Rashid et al., 2018; Alenezi et al., 2024).

In conclusion, cases of botulism associated with cosmetic BoNT-A injections are increasing, among which respiratory failure and MDR infection are emerging clinical challenges. Early tracheostomy might decrease the incidence of MDR among those who need mechanical ventilation. Health authorities should take measures to strengthen supervision on the cosmetic injections with BoNT-A.

## Data Availability

The original contributions presented in the study are included in the article/supplementary material, further inquiries can be directed to the corresponding authors.
